# Striatal Cholinergic Interneurons Display Activity-Related Phosphorylation of Ribosomal Protein S6

**DOI:** 10.1371/journal.pone.0053195

**Published:** 2012-12-28

**Authors:** Jesus Bertran-Gonzalez, Billy C. Chieng, Vincent Laurent, Emmanuel Valjent, Bernard W. Balleine

**Affiliations:** 1 Behavioural Neuroscience Laboratory, Brain and Mind Research Institute, The University of Sydney, New South Wales, Australia; 2 Institut de Génomique Fonctionnelle, Inserm U661, CNRS UMR 5203, Montpellier, France; INSERM/CNRS, France

## Abstract

Cholinergic interneurons (CINs) provide the main source of acetylcholine to all striatal regions, and strongly modulate dopaminergic actions through complex regulation of pre- and post-synaptic acetylcholine receptors. Although striatal CINs have a well-defined electrophysiological profile, their biochemical properties are poorly understood, likely due to their low proportion within the striatum (2–3%). We report a strong and sustained phosphorylation of ribosomal protein S6 on its serine 240 and 244 residues (p-Ser^240–244^-S6rp), a protein integrant of the ribosomal machinery related to the mammalian target of the rapamycin complex 1 (mTORC1) pathway, which we found to be principally expressed in striatal CINs in basal conditions. We explored the functional relevance of this cellular event by pharmacologically inducing various sustained physiological activity states in CINs and assessing the effect on the levels of S6rp phosphorylation. Cell-attached electrophysiological recordings from CINs in a striatal slice preparation showed an inhibitory effect of tetrodotoxin (TTX) on action potential firing paralleled by a decrease in the p-Ser^240–244^-S6rp signal as detected by immunofluorescence after prolonged incubation. On the other hand, elevation in extracellular potassium concentration and the addition of apamin generated an increased firing rate and a burst-firing activity in CINs, respectively, and both stimulatory conditions significantly increased Ser^240–244^-S6rp phosphorylation above basal levels when incubated for one hour. Apamin generated a particularly large increase in phosphorylation that was sensitive to rapamycin. Taken together, our results demonstrate for the first time a link between the state of neuronal activity and a biochemical signaling event in striatal CINs, and suggest that immunofluorescence can be used to estimate the cellular activity of CINs under different pharmacological and/or behavioral conditions.

## Introduction

The striatum is a large, subcortical structure involved in diverse cognitive and sensory-motor functions that, due to its massive glutamatergic and dopaminergic inputs from cortical, thalamic and midbrain regions, is regarded as the primary input to the basal ganglia [1]. The cytoarchitecture of the striatum is very well described and is almost entirely (∼95%) composed of GABAergic medium-sized spiny neurons (MSNs) subdivided into two subpopulations according to their projection targets and their selective expression of D1 and D2 dopamine receptor subtypes [2]. The remaining ∼5% of neurons are GABAergic and cholinergic interneurons (CINs) [3], [4]. CINs provide the main source of acetylcholine in the striatum [5] and, although they represent only 2–3% of the striatal neurons, they ramify very widely to generate amongst the highest cholinergic activity in the brain [6]. Morphologically, CINs have very large somata, widespread aspiny dendritic trees and extensive axonal fields covering large regions of the striatum [7]. Electrophysiological properties of CINs have been thoroughly investigated, and are characterized by their intrinsic tonic firing activity, when devoid of all their synaptic inputs [8], as well as a slow, regular firing rate with long duration action potentials and large afterhyperpolarizations [3], [7], [9]. However, although they mostly fire in this tonic mode, CINs are also capable of expressing a variety of firing patterns, some of which may overlap with those of the MSNs [8]. Acetylcholine released by CINs affects all striatal neurons, acting on nicotinic and muscarinic receptors expressed post-synaptically, or modulating these receptors expressed pre-synaptically on afferent terminals impinging on MSNs [10], [11].

Given the functional importance of the striatum and the contribution of CINs to those functions, it is important to be able rapidly and reliably to establish the level of activity of CINs under each striatal-dependent functional condition, Intracellular signaling responses triggered specifically in striatal CINs remain largely unexplored, however, due to the technical limitations that their low numbers in the striatal tissue cause for molecular, biochemical and electrophysiological studies [12]. Here we describe a means of estimating functionally relevant changes in CIN activity based on fluctuations in phosphorylation levels of the ribosomal protein S6 (S6rp) assessed by immunofluorescence. S6rp is an integrant of the ribosomal complex localized in the interface between the 40S and 60S subunits [13]. Although still debated, S6rp has been functionally implicated in the stimulation and/or inhibition of certain types of mRNA translation, as well as in the regulation of cell size and cell proliferation in different cellular systems [14], [15]. Importantly, S6rp is phosphorylated by ribosomal protein S6 kinases (S6K1 and 2), major downstream effectors of the mammalian target of rapamycin complex 1 pathway (mTORC1 pathway) [15], [16]. In eukaryotes, this phosphorylation occurs in a train of evolutionary conserved c-terminal serine residues between Ser^235^ and Ser^247^, and has been shown to be functionally relevant for cell size and glucose homeostasis regulation [14]. In the brain, strong regulation of S6rp phosphorylation has been recently described in the two populations of striatal projection MSNs after several dopaminergic pharmacological manipulations [17]–[19], and this same phosphorylation event has been recently used to capture translating ribosomes from activated neurons in response to a variety of stimuli [20].

Here, through a series of physiological and confocal immunofluorescence studies on striatal tissue, we report a strong and sustained phosphorylation of different c-terminal serine residues of S6rp specifically occurring in striatal CINs in basal conditions. This phosphorylation paralleled the state of physiological activity of CINs; the S6rp phosphorylation signal was markedly decreased when neuronal firing was pharmacologically prevented for one hour, and increased by sustained pharmacological stimulation of neuronal firing in a rapamycin-dependent manner. Our results demonstrate a link between neuronal activity and molecular signaling occurring in striatal CINs, and suggest a relatively straightforward means of estimating function specifically in this striatal population.

## Materials and Methods

### Ethics Statement

Long-Evan rats used in this study were obtained from Monash University Animal Research Platform. They were housed in plastic boxes (two rats per box) located in a climate- controlled colony room and were maintained on a 12 h light/dark cycle with food and water *ad libitum* until the time of experimentation. The Animal Ethics Committee at the University of Sydney approved all experimental procedures.

### Brain slice preparation

Male rats (4–8 weeks old) were sacrificed under deep anaesthesia by isoflurane inhalation (4% in air), and the brain was rapidly perfused with oxygenated cold sucrose buffer (transcardial 1 min) and dissected. Coronal brain slices (300 µm thick) containing the posterior dorsomedial striatum were cut using a vibratome (Leica Microsystems VT1200S, Germany) in the sucrose buffer containing (in mM): 241 sucrose, 28 NaHCO_3_, 11 glucose, 1.4 NaH_2_PO_4_, 3.3 KCl, 0.2 CaCl_2_, 7 MgCl_2_. Slices were hemisected at midline and maintained at 33°C in a submerged chamber containing physiological saline with composition (in mM): 126 NaCl, 2.5 KCl, 1.4 NaH_2_PO_4_, 1.2 MgCl_2_, 2.4 CaCl_2_, 11 glucose and 25 NaHCO_3_, and equilibrated with 95% O_2_ and 5% CO_2_.

### Electrophysiological recording

After equilibration for 1 h, slices were transferred to a recording chamber and visualized under an upright microscope (BX50WI, Olympus, Shinjuku, Japan) using differential interference contrast (DIC) Dodt tube optics, and superfused continuously (1.5 ml/min) with oxygenated physiological saline at 33°C. Cell-attached and whole-cell patch-clamp recordings were made using electrodes (2–5 MΩ) containing internal solution (in mM): 115 K gluconate, 20 NaCl, 1 MgCl_2_, 10 HEPES, 11 EGTA, 5 Mg-ATP, and 0.33 Na-GTP, pH 7.3, osmolarity 285–290 mOsm/L. Biocytin (0.1%, Sigma-Aldrich, St. Louis, MO) was routinely added to the internal solution for marking the sampled neurons during whole-cell recording. Data acquisition was performed with an Axopatch 200A amplifier (Molecular Devices, Sunnyvale, CA), connected to a Macintosh computer and interface ITC-16 (Instrutech, Long Island, NY). In cell-attached mode, action potentials were sampled at 10 kHz (low pass filter 5 kHz) and whole-cell currents were sampled at 5 kHz (low pass filter 2 kHz, Axograph X, Axograph, Berkeley, CA). Whole-cell recordings were established immediately following data collection in cell-attached mode. Stock solutions of all drugs were diluted to working concentrations in the extracellular solution (as indicated below) immediately before use and applied by continuous superfusion. Data from cell-attached and whole cell recordings were only included in analyses if (1) the neurons appeared healthy under DIC on monitor screen, i.e. showing smooth even cell membrane texture and integrity without visible nucleus, (2) cholinergic interneurons were spontaneously active during cell-attached recording, (3) action potential amplitudes were at least 70 mV after establishing whole-cell recording mode, and (4) neurons demonstrated physiological characteristics of cholinergic interneurons such as presence of hyperpolarization-activated cation current Ih but no plateau low-threshold spiking [3], to ensure that only highly viable neurons were included.

### Drugs and drug incubation in slices

Working drug concentrations for incubations were tetrodotoxin (TTX, 1 µM, Ascent Scientific, Bristol, UK), apamin (100 nM, Sigma, St Louis, MO; Abcam, Cambridge, MA) and rapamycin (1 µM with 0.02% DMSO, Santa Cruz Biotechnology Inc., Santa Cruz, CA). A cocktail of synaptic blockers was applied in some experiments, combining picrotoxin (100 µM, Sigma-Aldrich, St. Louis, MO), CNQX disodium and DL-AP5 (10 µM and 100 µM, respectively, Ascent Scientific, Bristol, UK). Drug incubations began immediately after slices were cut and hemisected as indicated above and lasted for 1 hour at 33°C. In all experiments, hemisected slices were counterbalanced, with half slice placed in a bath with vehicle solution (vehicle) and the other half in an identical bath containing the experimental drugs. Slices were then fixed overnight at 4°C in 4% paraformaldehyde/PBS containing 0.2% sodium fluoride (NaF) to inhibit remaining phosphoseryl phosphatase activity. Individualized sections were rinsed 3 times in Tris-buffered saline with NaF (TBS-NaF; 0.25 M Tris, 0.5 M NaCl and 0.1 mM NaF, pH 7.5) and permeabilized by incubation in 0.3% Triton X-100 in TBS-NaF for 5–6 hours. Post-permeabilization treatments are equal to those indicated below.

### Drug treatments *in vivo*

The same sets of drugs with adjusted concentrations were bilaterally delivered intrastriatally through stereotaxic surgery. In all rats, each brain side received either a control solution containing synaptic blockers (picrotoxin, 150 µM; CNQX disodium, 1 mM; DL-AP5, 1 mM in 0.9% NaCl with 0.02% DMSO) or the same solution plus the experimental drugs (apamin, 100 nM and/or rapamycin, 1 µM). Brain sides were counterbalanced in each group. At the time of surgery, 12-weeks old male Long-Evans rats weighted between 320 and 360 grams. They were anaesthetized with isoflurane (5% for induction and 2–3% for maintenance) and placed in a stereotaxic frame (David Kopf Instruments, Tujunga, CA). An incision was made to expose the scalp and the incisor bar was adjusted to align bregma and lambda to the same horizontal plane. Holes were drilled into the skull above the dorsal striatum (anteroposterior: +1, mediolateral: ±2.8, dorsoventral: −5.5, coordinates are in millimeters relative to bregma) [21]. Drugs were infused using a 1-µL microsyringe (Hamilton Neuros, Hamitlon, Reno, NV) connected to a microinfusion pump (Harvard Apparatus, Holliston, MA). A total volume of 0.5 µL was delivered in each hemisphere at a rate of 0.05 µL/min. The syringe was left in position for 2 additional minutes to allow for diffusion of drugs. After completion of drug infusion, rats were maintained under isoflurane anesthesia for 20 minutes and processed for transcardial fixation as indicated below.

### Transcardial fixation and sectioning

Rats were rapidly anaesthetized with sodium pentobarbital (300 mg/kg i.p., Virbac Pty. Ltd., Australia) and transcardially perfused with 4% paraformaldehyde in 0.1 M sodium phosphate buffer (pH 7.5). Brains were post-fixed overnight in the same solution and stored at 4°C. Coronal sections (30 µm, around +1 mm from bregma) [21] were cut with a vibratome (Leica Microsystems VT1000, Germany) and stored at −20°C in a solution containing 30% ethylene glycol, 30% glycerol and 0.1 M sodium phosphate buffer, until they were processed for immunofluorescence.

### Immunofluorescence

Individualized free-floating sections were rinsed in Tris-buffered saline with NaF (TBS-NaF; 0.25 M Tris, 0.5 M NaCl and 0.1 mM NaF, pH 7.5), incubated for 5 min in TBS-NaF containing 3% H_2_O_2_ and 10% methanol, and then rinsed 10 min three times in TBS-NaF. After 20 min incubation in 0.2% Triton X-100 in TBS-NaF, sections were rinsed three times in TBS-NaF again. DARPP-32 (D-32), choline acetyltransferase (ChAT) and the double phosphorylated form of S6 ribosomal protein (p-Ser^235–236^-S6rp or p-Ser^240–244^-S6rp) were simultaneously detected through incubation with combined purified mouse anti-DARPP-32 (1∶300, #611520, BD Biosciences, San Jose, CA), polyclonal goat anti-ChAT (1∶500, #AB144P, Millipore, Billerica, MA) and polyclonal rabbit anti-p-Ser^235–236^-S6rp or anti-p-Ser^240–244^-S6rp (1∶300, #2211 and #2215, Cell Signaling Technology, Beverly, MA) diluted in TBS-NaF (4°C, overnight). Sections were then rinsed 10 min in TBS-NaF three times and incubated 60 min with combined donkey anti-mouse Alexa 647-coupled, donkey anti-goat Alexa-594-coupled and donkey anti-rabbit Alexa-488-coupled secondary antibodies (Life Technologies, Carlsbad, CA) diluted 1∶400 in TBS. Sections were rinsed four times 10 min in TBS before mounting in Vectashield fluorescence medium (Vector laboratories, Burlingame, CA).

### Fluorescence analysis

All images were obtained using sequential laser scanning confocal microscopy (Zeiss LSM 710, Carl Zeiss AG, Oberkochen, Germany; FV300 and FV1000, Olympus, Shinjuku, Japan). In the triple immunofluorescence study, phospho-Ser^235–236^ and phospho-Ser^240–244^ signal intensities were compared in MSNs and CINs coexisting in the same tissue region. In 425.1 µm^2^ images (resolution: 2.409 pixels/µm), a region of interest (ROI) was defined containing 40 clear D-32 immunoreactive neurons (MSN mask) and 13 ChAT immunoreactive neurons (CINs mask) in tissue from 2 different rats. For each phospho-serine pair, fluorescence intensity (mean gray value), defined by the MSN and the CIN masks, was measured in the p-S6rp superimposed image. In studies of individualized cholinergic interneurons, all clear ChAT-immunoreactive neurons in dorsal striatal regions (10–15 neurons per hemisphere) were detected using the confocal microscope with a 60X objective (UPFL 60X oil) and centered in the acquisition area. Focal plane with optimal ChAT immunoreactivity was determined in channel 2 (Ch02, HeNe green laser). Sequential 58.93 µm^2^ images (optical magnification: 60X; digital zoom: 4X; resolution: 17.378 pixels/µm) were obtained for ChAT signal (Ch02, HeNe green laser intensity usually: 35.0%; PMT: 720v; offset: 2%) and corresponding p-Ser^240–244^-S6rp signal (Ch01, Ar laser intensity usually: 25.5%; PMT: 740v; offset: 2%) with a Kaplan filter (5 averaging scans). Raw 16-bit images were then analyzed using Open Source ImageJ software (MacBiophotonics upgrade v. 1.43u, Wayne Rasband, National Institutes of Health, USA). Fluorescence intensity (FI) of the phospho-S6rp signal in each neuron was studied by defining an image mask in Ch02 (ROI1: somatic area; ROI2: background in 15 um^2^ in the vicinity of the soma) and superimposing it to Ch01. The mean gray value of the pixels contained in ROI1 and ROI2 was obtained for Ch01, and p-S6rp signal was defined as ROI1 minus ROI2. Prior to all quantifications, all image files in each experiment were randomly renumbered using a MS Excel plug-in (Bio-excel2007 by Romain Bouju, France). A pseudo-color palette highlighting intensity of fluorescence (16-color LookUp Table, display range: 0–4096) was applied to representative neurons.

In post-fixation immunofluorescence assays performed in live tissue sections, a notable level of variability in the general immunofluorescence signal is often observed between subjects in the same experiment. This may be due to the intrinsic variability generated during the process of *in vitro* brain slice preparation and tissue viability during the incubation time prior to fixation. To overcome these problems, all experiments were conducted following a within-subjects design, such that each experimental subject was also its own control. In order to make individuals comparable, despite the effects showing statistical significance when raw mean gray values were considered, we normalized our data across individuals by dividing each individual FI value by the mean FI in the control sections (fluorescence arbitrary units [a.u.]; all controls tend to 1.0). Data are presented as dot plots where per neuron values as well as mean values are indicated. Quantified neurons from each subject are presented in a different color. Statistical comparisons were made using Prism (v. 5.0, GraphPad Software, Inc., San Diego, CA). We used unpaired t-test for 2 group comparisons and one-way ANOVA with Bonferroni's post-hoc test for 3 or more group comparisons. Statistical comparisons were considered significant if p<0.05.

## Results

### Striatal cholinergic interneurons show phosphorylation of ribosomal protein S6 in basal conditions

**Figure 1:**
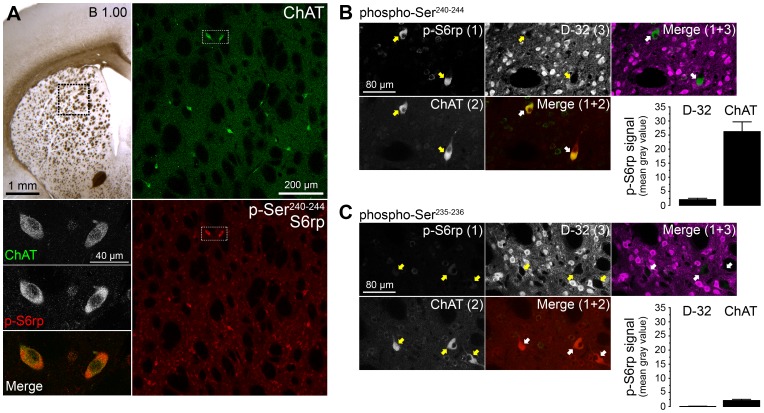
Cholinergic interneurons (CINs) in the striatum show selective phosphorylation of S6rp in basal conditions. (A) Low magnification image from a transcardially-fixed naïve rat brain section showing the striatal level analyzed in the present study. Inset corresponds to magnified right panels: low magnification images of the dorsal striatum double-stained with choline acetyltransferase (ChAT, green) and phosphorylated ribosomal protein S6 at Serine 240 and 244 residues (p-S6rp, red) showing substantial co-localization. Insets (bottom-left) are high magnification confocal images showing ChAT and p-S6rp immunoreactivity in two adjacent CINs. (B, C) Confocal sections of rat striatal tissue stained for two different pairs of phospho-serine residues at the C terminus of S6rp (B, Ser^240–244^ and C, Ser^235–236^, green) combined with ChAT (red) and DARPP-32 (D-32, magenta). Arrows indicate ChAT-immunoreactive neurons. (B, C; bottom right graphs) Intensity fluorescence study of p-Ser^240–244^ (B) and p-Ser^235–236^ (C) S6rp signal contained in MSNs (D-32 immunoreactive) and CINs (ChAT immunoreactive) in triple-stained sections. Data are mean ± SEM; n = 2 rats; 40 MSNs and 13 CINs quantified per group.

Recent studies report that the different populations of striatal projection MSNs express strong and sustained phosphorylation of S6rp at different c-terminal serine residues in response to several dopaminergic pharmacological manipulations [17]–[19]. Interestingly, control, as well as stimulated animals in these studies displayed high levels of S6rp phosphorylation in a sparse population of striatal neurons, which were identified as putative cholinergic interneurons (CINs) due to their large size [17]. We directly confirmed the identity of the neurons showing S6rp phosphorylation in non-stimulated rats through a confocal microscopy study on striatal tissue (Figure 1). In the dorsal striatum, we simultaneously detected the double phosphorylated form of S6rp (p-Ser^240–244^) and choline acetyltransferase (ChAT), a reliable marker of CINs [22], through double immunofluorescence. As expected, the level of co-localization between ChAT-immunoreactive neurons and neurons expressing high levels of p-Ser^240–244^ signal was almost complete, although a basal phosphorylation signal was also detected in other striatal neurons (Figure 1A). Higher magnification analysis in ChAT neurons revealed that the p-S6rp signal was mostly distributed across the perinuclear and somatic compartments, consistent with the preferential distribution of ribosomal complexes (Figure 1A, bottom-left panels). Despite both ChAT and p-S6rp being similarly distributed in these compartments, the co-localization signal within the cell soma remained low, excluding fluorescence overlapping artifact of acquisition (Figure 1A, bottom-left panels).

Previous studies found that phosphorylation of S6rp occurs in different sets of c-terminal serines, which are generally modulated by mTORC1-dependent signaling events, although alternative molecular regulation has been recently reported [18], [23]. We, therefore, next compared the levels of phosphorylation in two different pairs of c-terminal serines (phospho-Ser^240–244^ and phospho-Ser^235–236^) expressed in different striatal populations of naïve rats (Figure 1B, C). Our immunofluorescence study revealed a clear phospho-Ser^240–244^ signal preferentially expressed in ChAT neurons (Figure 1B), in contrast to a much weaker signal recorded for the phospho-Ser^235–236^ residues (Figure 1C). For both serine pairs, we compared the signal recorded in CINs (ChAT immunoreactive) with that of MSNs (DARPP-32 immunoreactive) [24] within the same focal plane. Despite the contrasting intensity of phospho-Ser^240–244^ over phospho-Ser^235–236^ signal, fluorescence quantification in CINs and MSNs clearly revealed a higher intensity signal in ChAT as compared to DARPP-32 immunoreactive neurons in both phospho-serine pairs (Figure 1B, C). Due to the better resolution provided by phospho-Ser^240–244^ S6rp immunofluorescence, we used detection of this phospho-serine pair in further experiments in this study (from now on referred to as phospho-S6rp in the text).

### Study of the physiological features of cholinergic interneurons and their levels of S6rp phosphorylation in striatal slices *in vitro*

**Figure 2:**
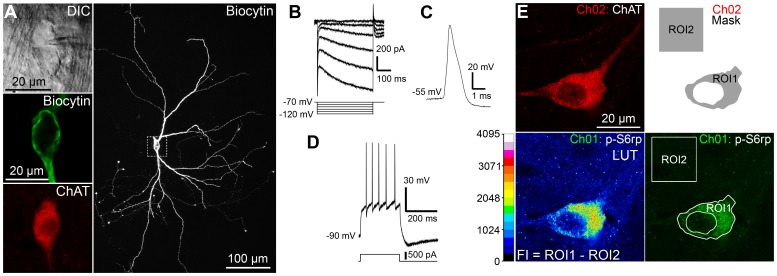
Cellular physiology and S6rp phosphorylation in CINs from viable striatal slices. (A) A representative striatal ChAT-immunoreactive neuron labeled with biocytin during electrophysiological recording. Visualization of the neuron after difference interference contrast (DIC) illumination (top left A), post hoc single optical scan of biocytin (middle left A) and ChAT labeling (bottom left A), and confocal Z stack projection of biocytin labeling at low magnification (20×, right A). (B–D) Cellular physiological characteristics of the neuron in A under whole-cell patch-clamp. Current-voltage relationship recorded by stepping the cell to various hyperpolarizing membrane potentials (B). Under current-clamp configuration, whole-cell action potential (C) and depolarization-triggered action potential firing (D) were routinely sampled for comparisons with known CIN cellular characteristics. (E) Quantification of p-S6rp in a ChAT-immunoreactive neuron from a viable striatal slice after overnight fixation and immunofluorescence. The neuron was identified under the microscope and a high magnification optical section was taken sequentially (Ch01: p-Ser^240–244^S6rp; Ch02: ChAT). Fluorescence intensity (FI) of the phospho-S6rp signal in each neuron was studied by defining an image mask in Ch02 (ROI1: somatic area; ROI2: background in 15 um^2^ in the vicinity of the soma) and superimposing it onto Ch01. The mean gray value of the pixels contained in ROI1 and ROI2 was obtained for Ch01, and FI was defined as ROI1 minus ROI2. A 16 pseudo-color palette (Lookup Table, LUT) applied in Ch01 highlights the intensity of p-S6rp fluorescence. The LUT scale defines 16-color intervals according to pixel gray values in a 16-bit image.

As the intracellular signaling leading to phosphorylation of S6rp has been related to translational regulation, cell-size maintenance and protein-dependent synaptic plasticity in different cellular systems [15], [25], [26], we hypothesized that the high level of S6rp phosphorylation signal expressed in CINs over other types of striatal neurons could be related to their intrinsic tonic firing activity expressed in basal conditions [3], [9]. To address this question, we designed a set of *in vitro* experiments, in which electrophysiological recordings and post-fixation immunofluorescence were performed in subsequent striatal sections (Figure 2). Although representing only 2–3% of the entire neuronal population in the striatum, selective *in vitro* electrophysiological sampling of CINs is relatively straightforward due to their larger soma, which is reliably identified under differential interference contrast illumination (DIC, Figure 2A). Twenty-five out of thirty-four presumed cholinergic interneurons were labeled with biocytin for *post-hoc* morphological validation (with eventual biochemical ChAT-immunoreactivity validation; Figure 2A), and all of them agreed with the morphological descriptions of striatal cholinergic interneurons, displaying a large multipolar or fusiform soma and long aspiny dendrites [27]. An extensive axonal tree was occasionally observed in some cells, although it depended on the degree of biocytin spread [3], [8] (Figure 2A). All cholinergic interneurons considered in this study, as assessed under DIC illumination (just before patching), were at least 2–3 times larger than nearby more abundant MSNs. We systematically showed in these neurons the characteristic physiological features of CINs, including the presence of hyperpolarization-activated cationic current (Ih, Figure 2B), the long duration of their action potentials (1.96±0.06 ms, range 1.19–2.76 ms, n = 34, Figure 2C) and a lack of low-threshold burst of spike discharge [3], [8], [9] (Figure 2D).

In parallel, striatal slices were maintained in an oxygenated ACSF bath for one hour, during which various pharmacological treatments modulating CIN activity were applied. After incubation, samples were fixed and processed for immunofluorescence to study the S6rp phosphorylation levels expressed in ChAT-immunoreactive neurons (Figure 2E). The robustness of this method lies in the accurate measurement of the phospho-S6rp immunofluorescence signal expressed in each CIN individually. Although more laborious, quantification of signal intensity on a per neuron basis was necessary in order to detect any possible biochemical activity change in CINs, since virtually all neurons expressed basal phospho-S6rp signal, making immunoreactive cell counts ineffective to measure the effect. To overcome this drawback, a single optical scan of each ChAT-immunoreactive neuron in the dorsal striatum (approximately 10–15 per hemisphere) was performed followed by sequential capture of their phospho-S6rp signal at a set laser intensity. We then measured the specific phospho-S6rp fluorescence intensity contained in the ChAT neuron soma (Figure 2E, see materials and methods section for details). Taken together, our parallel physiological and immunofluorescence studies in striatal slices *in vitro* provided a good indication of the physiological responses and the intracellular biochemical activity expressed by CINs in response to extended pharmacological treatments.

### Sustained reduction of action potential firing by tetrodotoxin incubation in cholinergic interneurons results in decreased phosphorylation of ribosomal protein S6

**Figure 3:**
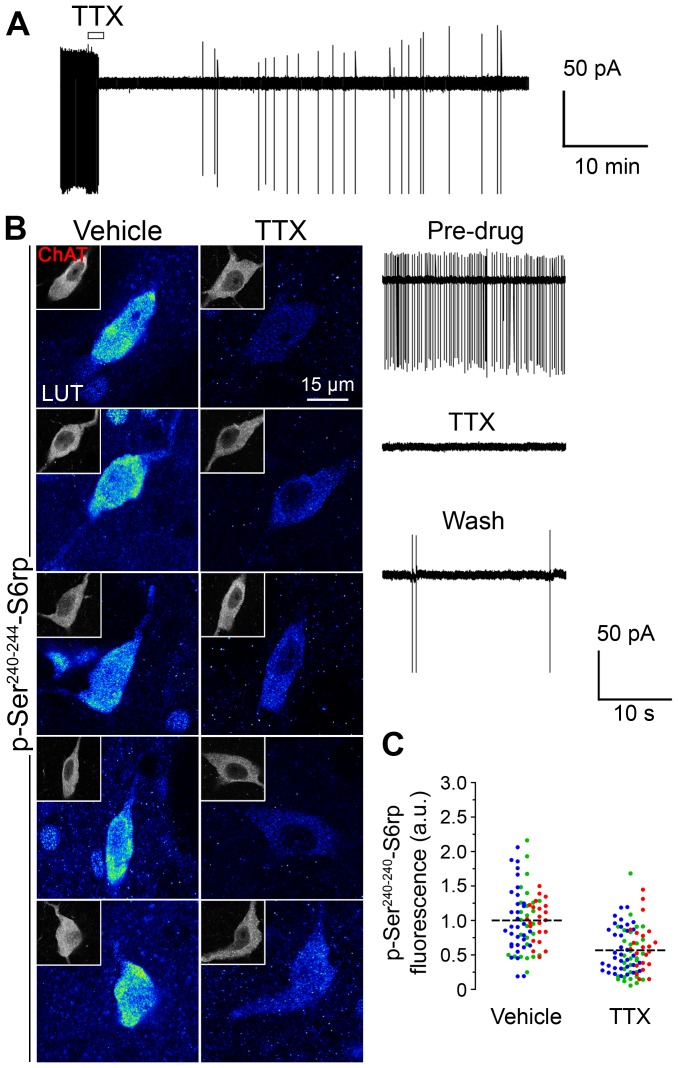
Tetrodotoxin (TTX) inhibits basal cellular activity and reduces S6rp phosphorylation in striatal CINs. (A) Cell-attached recording of a striatal cholinergic interneuron depicting TTX (100 nM) inhibition of spontaneous action potential firing. The open bar indicates time of TTX application. The bottom traces show an expanded time scale (n = 3 neurons). (B) High-magnification confocal images of 5 different striatal CINs (ChAT-immunoreactive, insets) and their corresponding p-Ser^240–244^-S6rp levels in slices incubated in physiological saline (vehicle) or plus TTX (1 µM). A 16 pseudo-color palette LUT highlights intensity of p-S6rp fluorescence. (C) Quantification of the p-S6rp signal in each striatal ChAT immunoreactive neuron after 1-hour incubation in control or TTX (1 µM). P-S6rp signal intensity for each neuron was calculated as in Figure 2E. In scatterplot, each dot corresponds to one neuron; each color corresponds to a different animal; dashed lines indicate the mean. Fluorescence values are normalized in arbitrary units (a.u.). Data were analyzed using unpaired Student *t-test*: p<0.0001; 81–88 quantified neurons per condition in 3 rats.

Blockade of voltage-gated sodium channels by tetrodotoxin (TTX) has been shown to prevent action potential firing in many neuronal types, including striatal CINs [9]. In the present study, we bath-applied 100 nM TTX to striatal slice preparations during cell-attached recordings on CINs, and the first action potential was observed at 8±3 min (n = 3) after washing, although full recovery was not achieved until >1 hr. We observed a strong inhibitory effect of TTX on spontaneous CIN firing in basal conditions (firing before TTX was 2.8±0.6 Hz, n = 3, Figure 3A). In the same slice preparations, 1 h incubation with TTX (1 µM) led to a concomitant reduction in the S6rp phosphorylation signal of ChAT-immunoreactive neurons (Figure 3B–C). These results show that the sustained suppression of CIN intrinsic firing caused by TTX through sodium channel inhibition can lead to biochemical signaling changes in proteins that are part of the ribosomal machinery.

### Sustained stimulation of cellular activity leads to increased phosphorylation of ribosomal protein S6 in cholinergic interneurons

**Figure 4:**
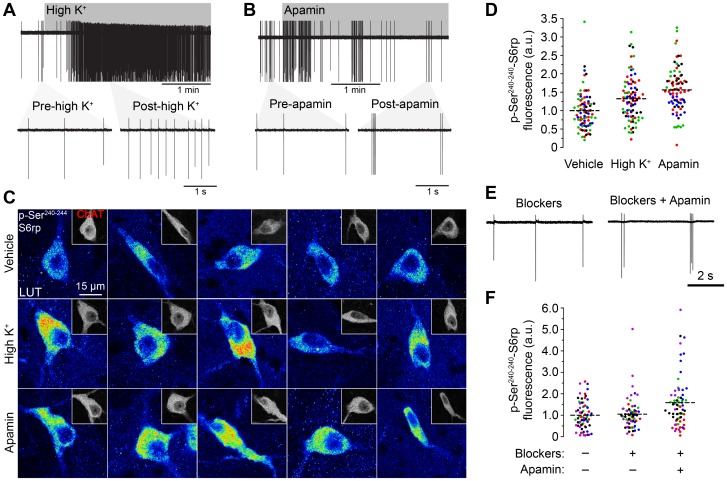
Different forms of cellular activity stimulation increase S6rp phosphorylation over basal levels in striatal CINs. (A) Cell-attached recording of a striatal CIN showing a strong increase in action potential firing after elevation of extracellular potassium concentration (High K^+^, from 2.5 mM in pre-condition to 11.5 mM in post-condition; n = 3 neurons). Gray shade indicates time of elevated K^+^ application. Bottom traces show an expanded time scale in pre- and post- K^+^ elevation. (B) Cell-attached recording of a striatal CIN showing typical burst-firing behavior after application of apamin (100 nM; n = 7 neurons). Gray bar indicates time of apamin application. Bottom traces show an expanded time scale in pre- and post- apamin conditions. (C) High-magnification confocal images of 5 different striatal CINs (ChAT-immunoreactive, insets) and their corresponding p-Ser^240–244^-S6rp levels in slices incubated in physiological saline (vehicle), high K^+^ (elevated extracellular K^+^ to 11.5 mM) or apamin (100 nM). A 16 pseudo-color palette LUT highlights intensity of p-S6rp fluorescence. (D) Quantification of p-S6rp signal in striatal ChAT immunoreactive neurons in each incubation condition. The p-S6rp signal intensity for each neuron was calculated as in Figure 2E. (E) Cell-attached recording of a CIN in the presence of synaptic blockers picrotoxin (Pic, 100 µM), CNQX (10 µM) and DL-AP5 (AP5, 100 µM). Application of apamin (100 nM) induced burst-firing responses as in A (n = 9 neurons). (F) Quantification of the p-S6rp signal in striatal ChAT immunoreactive neurons in each incubation condition. Application of synaptic blockers (Block) did not alter baseline p-S6rp signal nor did it inhibit the stimulatory effects of apamin (100 nM). In scatterplots (D and F), each dot corresponds to one neuron; each color corresponds to a different animal; dashed lines indicate the mean. Fluorescence values are normalized in arbitrary units (a.u.). Data were analyzed with one-way ANOVA: D: Effect of drug: F_(2,247)_ = 20.11, p<0.0001; Bonferroni post hoc: Vehicle vs. High K^+^: p<0.001; Vehicle vs. Apamin: p<0.001; High K^+^ vs. Apamin: p<0.05; 81–87 neurons quantified per condition in 4 rats. F: Effect of drug: F_(2,201)_ = 9.503, p<0.0001; Bonferroni post hoc: Vehicle vs. Block+Apamin: p<0.001; Block vs. Block+Apamin: p<0.01; 66–70 quantified neurons per condition in 5 rats).

We next assessed the extent to which S6 phosphorylation in CINs was altered by two different types of pharmacological excitation. We tested the effect of elevated extracellular potassium concentration (high K^+^), which is known to increase neural excitability, as well as the effect of SK channel blockade using apamin, a treatment that induces robust burst-firing activity in CINs [9] (Figure 4). Regular firing and burst-firing were measured using mean action potential frequency (Hz) and variance of instantaneous action potential frequency (sq Hz), respectively. In cell-attached recordings on identified CINs, elevation of extracellular potassium (from 2.5 to 11.5 mM) dramatically increased firing of action potentials (0.8±0.4 Hz at 2.5 mM vs. 2.3±0.6 Hz at 11.5 mM, mean ± SEM, n = 6, paired-t test, p<0.05, Figure 4A). The variance of instantaneous action potential frequency was not significantly changed by elevated potassium treatment (0.1±0.1 sq Hz at 2.5 mM vs. 0.9±0.5 sq Hz at 11.5 mM, mean ± SEM, n = 6, paired-t test, p>0.05,). On the other hand, application of apamin (100 nM) induced clear burst-firing responses in all recorded cells, as indicated by rapid spiking periods alternating with silent activity (Figure 4B) consistent with previous reports [9]. The variance of instantaneous action potential frequency was significantly increased (indicative of burst-firing) by apamin (0.5±0.3 sq Hz before vs. 17±5 sq Hz after apamin, mean ± SEM, n = 5, paired-t test, p<0.05) whilst mean frequency was unchanged (0.8±0.4 Hz before vs. 0.7±0.2 Hz after apamin, mean ± SEM, n = 5, paired-t test, p>0.05). The basal action potential frequency (0.8±0.4 Hz, n = 6 for high K^+^ vs. 0.9±0.4 Hz, mean ± SEM, n = 5 for apamin, unpaired-t test, p>0.05) and variance of instantaneous frequency (0.1±0.1 sq Hz, n = 6 for high K^+^ vs. 0.5±0.3 Hz, n = 5 for apamin, mean ± SEM, unpaired-t test, p>0.05) did not differ between high K^+^ and apamin groups. Interestingly, the immunofluorescence analysis of slices incubated with the same treatments for 1 hour showed a dramatic increase in phosphorylation levels of S6rp in ChAT immunoreactive neurons in both stimulation conditions (Figure 4C). Fluorescence quantification confirmed a significant increase of CIN S6rp phosphorylation after both high K^+^ and apamin incubations (Figure 4D). Importantly, SK channel blockade by apamin induced the strongest effects on CIN S6rp phosphorylation, which were significantly higher than those of high K^+^. Based on previous proposals that SK channel-dependent burst-firing activity is critical for striatal physiology [28], [29], and the fact that SK channel blockade induced the strongest effects on CIN S6rp phosphorylation, we used apamin stimulation in further experiments.

Excitatory and inhibitory synaptic inputs onto CINs are regulated by muscarinic and nicotinic receptors in the striatum [29]–[31]. Since our goal here was to assess the specific role of intrinsic postsynaptic responses of CINs and their influence on S6rp phosphorylation, we used pharmacology to isolate these neurons from their glutamatergic and GABAergic inputs [8]. To prevent presynaptically released glutamate and GABA acting on ionotropic AMPA, NMDA and GABA_A_ receptors located on CINs, slices were incubated in a cocktail of synaptic blockers: picrotoxin (100 µM), CNQX (10 µM) and DL-AP5 (100 µM). Cell-attached recordings showed, as previously reported, that incubation with synaptic blockers did not modify the firing of CINs in basal conditions (1.6±0.5 Hz before and 1.5±0.3 Hz after blockers, n = 9, paired-t test, p>0.05; [8]), and their presence did not prevent the burst-firing behavior induced by apamin (Figure 4E). Importantly, ChAT-immunoreactive neurons in slices incubated with or without synaptic blockers did not show any difference in their phospho-S6rp levels, and the addition of apamin to the solution continued to generate increased phosphorylation signal (Figure 4F). Taken together, these findings suggest that different patterns of postsynaptic physiological activity in CINs generated by persistent pharmacological stimulation can differentially influence the state of S6rp phosphorylation.

### The mTORC1 signaling pathway is involved in the biochemical but not the physiological responses of cholinergic interneurons to apamin

**Figure 5:**
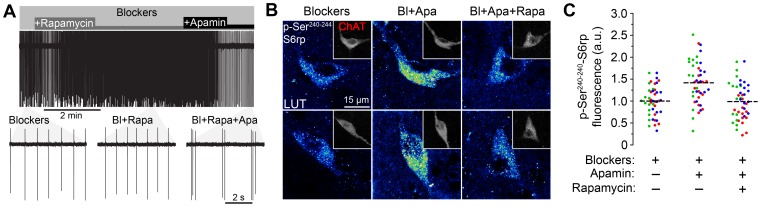
Rapamycin prevents apamin-stimulated S6rp phosphorylation but not cellular activity in striatal CINs. (A) Cell-attached recording of a CIN in the presence of synaptic blockers picrotoxin (Pic, 100 µM), CNQX (10 µM) and DL-AP5 (AP5, 100 µM) showing changes in firing after application of rapamycin (dark gray) and apamin (black). As shown in the bottom traces (n = 3 neurons), application of rapamycin (Rapa, 1 µM) did not alter spontaneous action potential firing nor did it affect the burst-firing response induced by final apamin application (Apa, 100 nM, n = 3 neurons). (B) High-magnification confocal images of representative striatal CINs (ChAT-immunoreactive, insets) and their corresponding p-Ser^240–244^-S6rp levels in slices incubated in the presence of synaptic blockers (Bl), plus 100 nM apamin (Bl+Apa) and plus 1 µM rapamycin (Bl+Apa+Rapa). (C) Quantification of the p-S6rp signal in striatal ChAT immunoreactive neurons in each incubation condition (intensity for each neuron was calculated as in Figure 2E). A 16 pseudo-color palette LUT highlights the intensity of p-S6rp fluorescence. In scatterplot, each dot corresponds to one neuron; each color corresponds to a different animal; dashed lines indicate the mean. Fluorescence values are normalized into arbitrary units (a.u.). Data were analyzed with one-way ANOVA (Effect of drug: F_(2,132)_ = 15.54, p<0.0001; Bonferroni post hoc: Blockers vs. Blockers+Apamin; p<0.001; Blockers+Apamin vs. Blockers+Apamin+Rapamycin; p<0.001; 45 quantified neurons per condition in 3 rats).

We next sought to study the molecular mechanisms that mediate the effects of apamin on S6rp phosphorylation in CINs. In many neuronal and non-neuronal systems, c-terminal serine residues of S6rp have been shown to be directly phosphorylated by S6K1 and 2, two ubiquitous kinases that are tightly regulated by the mTORC1 complex [15]. A very useful drug to assess mTORC1 activity is rapamycin, which effectively inhibits mTORC1 in different cellular systems by targeting the adaptor protein Raptor [25]. Recently, however, alternative molecular signaling pathways leading to S6rp phosphorylation independently of mTORC1 have been described in response to different pharmacological stimuli in striatopallidal MSNs [18]. We sought, therefore, to assess whether the different cellular effects mediated by apamin in CINs were dependent on the mTORC1 pathway. In our cell-attached recording experiments, rapamycin (1 µM) did not affect basal firing rate expressed by CINs, and failed to prevent apamin-induced burst-firing activity (Figure 5A). The action potential frequencies (mean ± SEM) were 1.2±0.3 Hz before, 1.3±0.5 Hz after rapamycin and 0.9±0.3 Hz after the addition of apamin, n = 3. The variance of instantaneous action potential frequencies (mean ± SEM) was 0.03±0.01 sq Hz before, 0.03±0.02 sq Hz after rapamycin and 44±36 sq Hz after the addition of apamin, n = 3. Interestingly, subsequent immunofluorescence studies in slices submitted to prolonged incubation showed that rapamycin (1 µM) prevented the increase of S6rp phosphorylation shown in CINs in response to apamin incubation (Figure 5B–C), pointing to the downstream involvement of the mTORC1 pathway in the mediation of the apamin-induced intracellular responses.

**Figure 6:**
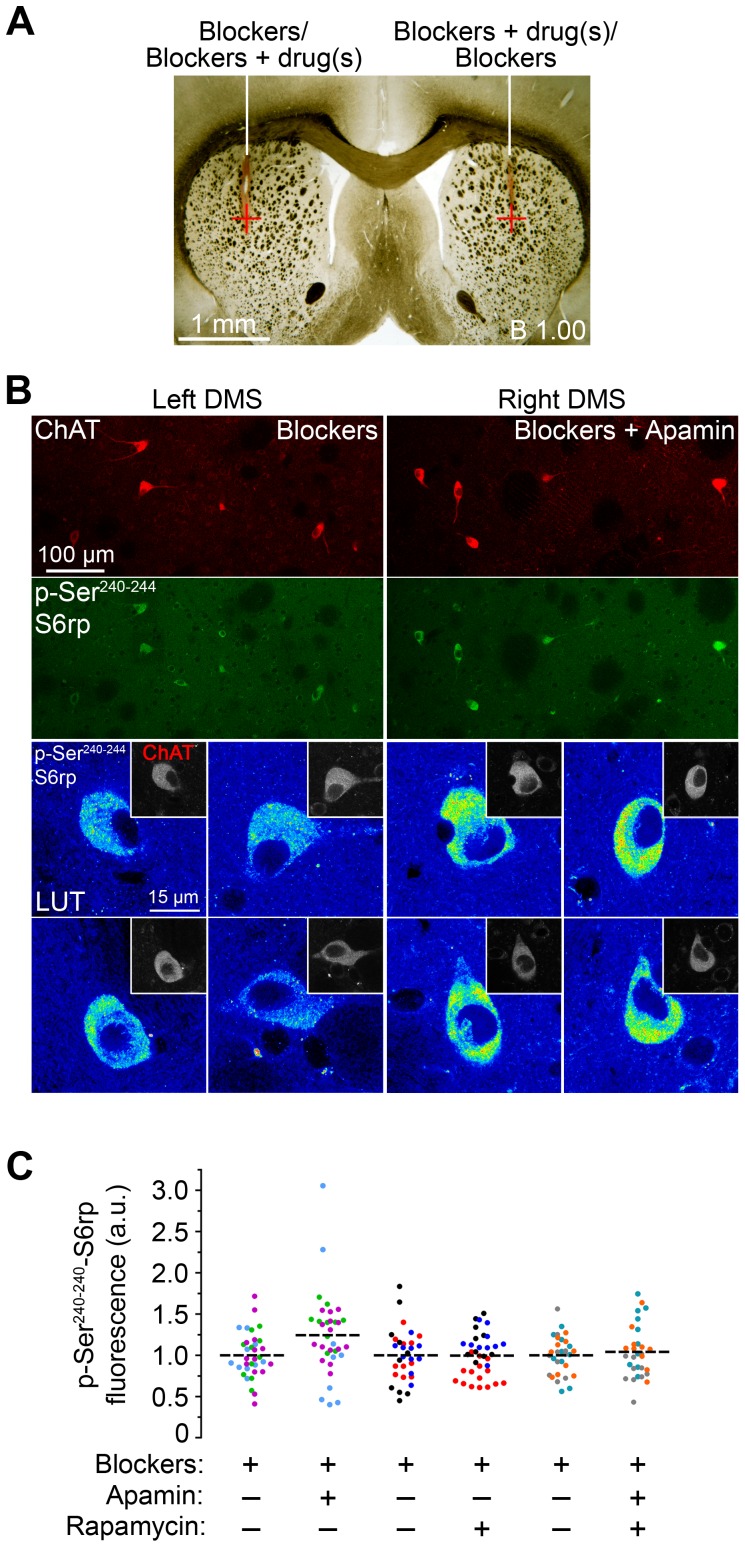
Effects of apamin and rapamycin on S6rp phosphorylation in CINs are reproduced *in vivo*. (A) Low magnification image of a rat brain section showing the targeted striatal region bilaterally (red cross). Rats received either synaptic blocker solution (blockers; picrotoxin, 150 µM; CNQX disodium, 1 mM; DL-AP5, 1 mM) or the same solution plus experimental drug(s) (apamin, 100 nM and/or rapamycin, 1 µM) in contralateral hemispheres. (B) Confocal images from a transcardially-fixed rat brain previously injected with blockers (left-side dorsal striatum, Left DMS) and blockers plus apamin (right-side dorsal striatum, Right DMS) showing double staining for ChAT and p-Ser^240–244^-S6rp. Top panels are low-magnification images showing several cholinergic interneurons in the same focal plane. Bottom panels are higher magnification images showing p-S6rp signal intensity in CINs from the left (blockers) and right (blockers + apamin) striata of the same animal. Insets show corresponding ChAT staining. (C) Quantification of p-S6rp signal in striatal ChAT immunoreactive neurons after each combination of injections (intensity for each neuron was calculated as in Figure 2E). A 16 pseudo-color palette LUT highlights the intensity of p-S6rp fluorescence. In the scatterplot, each dot corresponds to one neuron; each color corresponds to a different animal; dashed lines indicate the mean. Fluorescence values are normalized into arbitrary units (a.u.). Data were analyzed with one-way ANOVA (Effect of drug: F_(5,188)_ = 2.932, p = 0.0142, Bonferroni post hoc: Blockers vs. Blockers+Apamin; p<0.01; 30–35 quantified neurons per condition in 9 rats).

The physiological response of striatal neurons relies heavily on afferent inputs from the cortex, thalamus and substantia nigra. In brain slices, however, these projections are largely severed, which may in some cases affect the normal functioning of the neurons. To partially overcome these limitations and confirm the effects reported above *in vitro*, we designed an *in vivo* experiment in which drugs were infused directly into the dorsal striatum of anaesthetized rats, followed by processing of phospho-S6rp and ChAT immunofluorescence (Figure 6). Rats received either a solution containing synaptic blockers (picrotoxin, 150 µM; CNQX disodium, 1 mM; DL-AP5, 1 mM) or this same solution plus the experimental drug(s) (apamin, 100 nM and/or rapamycin, 1 µM) infused into each hemisphere in a counterbalanced manner (Figure 6A). Twenty minutes after drug infusion, confocal analysis of ChAT and phospho-S6rp revealed striking co-localization levels, with the phospho-S6rp signal outside of CINs being almost undetectable, likely due to the effect of the synaptic blockers on MSNs (Figure 6B, top panels). Importantly, S6rp phosphorylation was strongly increased in ChAT-immunoreactive neurons in the apamin-injected hemispheres (Figure 6B, bottom panels). Fluorescence quantification revealed a significant increase of S6 phosphorylation in CINs induced by apamin, an effect that was completely prevented by rapamycin (Figure 6C). Importantly, rapamycin alone did not further reduce the basal phospho-S6rp signal (Figure 6C), suggesting that other molecular mechanisms may be maintaining phosphorylation levels in basal conditions. Together, these results confirm and extend the pharmacological effects on S6rp phosphorylation in CINs *in vitro* described above.

## Discussion

In this study, we identified a phosphorylated form of S6rp, an integrant of the ribosomal complex, specifically expressed in cholinergic interneurons in basal conditions. Two different pairs of c-terminal serines appeared to be up-regulated in these neurons as compared to projection MSNs, although the Ser^240–244^ pair was found to provide a higher phosphorylation signal. Through electrophysiological and confocal immunofluorescence studies, we found that prolonged silencing of the activity of these neurons using the selective sodium channel blocker tetrodotoxin led to a marked decrease of S6rp phosphorylation, whereas drugs that increased the activity of CINs in different ways stimulated this phosphorylation above basal levels when incubated for one hour. Elevated concentrations of extracellular potassium, which caused a marked increase of CIN firing, induced a significant increase of S6rp phosphorylation above basal levels after extended application. Incubation with apamin, a selective SK channel blocker that stimulated activity by generating a burst-firing pattern in CINs, induced especially high levels of S6rp phosphorylation, and this effect was maintained after the synaptic isolation of these neurons. We also provided evidence for the involvement of the mTORC1 pathway only in the biochemical effects of apamin, since the mTORC1 complex inhibitor rapamycin prevented the increase in S6rp phosphorylation triggered by apamin incubation, but did not alter apamin-induced burst-firing activity.

Cholinergic interneurons have been extensively studied electrophysiologically, have clear morphological features and, therefore, the physiological patterns leading to their identification have been accurately described [3], [7]–[9]. However, electrophysiological recordings are limited, often to just a few neurons, and are impractical when large numbers of neurons need to be sampled, such as in functional histological studies after *in vivo* pharmacological or behavioral manipulations. For these kinds of study, neuronal activity markers – involving the detection of phosphorylation pathways or immediate-early gene expression – have been shown to be very useful in some cases, although these events are not ubiquitously activated in all neurons alike. In the striatum, the specific biochemical and histochemical study of CINs is hindered by the low proportion of these neurons in the tissue, and very little evidence for exclusive molecular regulation of this neuronal type exists [32]. Recent studies have, however, reported new molecular regulators that appear to be especially recruited by CINs in the ventral striatum to modulate depression-like effects, although direct links between the activity state of CINs and the regulation of these pathways remain to be elucidated [33]. More recently, an important G-protein-coupled receptor kinase (GRK2) has been found to be especially enriched in CINs compared to other types of neuron in the striatum, which suggests that important specific signaling events may be selectively occurring in this cell type [34]. In our study, the identification of a phosphorylation event in a protein integrant of the ribosomal machinery specifically occurring in CINs in basal conditions provides a potential marker reflecting the tonic activity state of these neurons.

Indeed, recent studies have examined the phosphorylation of S6rp in different populations of projection MSNs in response to several dopaminergic conditions. Santini et al. (2009) reported that, in dopamine-depleted striata, L-DOPA strongly increased phosphorylation of S6rp (both at Ser^240–244^ and Ser^235–236^ residues) in striatonigral MSNs. On the other hand, we previously showed that haloperidol, an antipsychotic drug blocking dopamine D2-receptors, strongly increased phosphorylation of Ser^235–236^-S6rp selectively in striatopallidal MSNs [18]. More recently, Gangarossa et al. (2012) reported strong S6rp phosphorylation selectively in striatonigral MSNs after treatment with a dopamine D1-receptor agonist. All pharmacological manipulations in these studies are known to boost the activity of the neurons concerned, thus giving a hint that phosphorylation of S6rp may be related to an increase in neuronal activity. Along similar lines, Knight et al. (2012) have very recently developed a method to capture translating ribosomes from discrete populations of activated neurons in brain homogenates that relies on this phosphorylation of S6rp [20]. All these studies reveal the potential of S6rp phosphorylation detection as a means to assess neuronal activity *in vivo*. In the present study, our ability to measure the extent of S6rp phosphorylation in CINs using immunofluorescence in striatal tissue paves the way for investigations into the functional roles played by these interneurons in complex pharmacological and behavioural paradigms *in vivo*.

Rapamycin is widely used as evidence for mTORC1 pathway involvement, attributed to its high specificity as an inhibitor of the mTORC1 complex [25]. Based on this, our results suggest that the mTORC1 pathway is involved in the increase of S6rp phosphorylation induced by apamin, as shown both in striatal slices and *in vivo*. However, rapamycin did not affect either basal intrinsic firing or apamin-induced burst-firing, suggesting that mTORC1 signaling is downstream of the apamin-mediated cell surface ion channel regulation of CINs. Interestingly, rapamycin directly injected into the striatum failed to reduce the basal S6rp phosphorylation levels expressed in CINs. These results suggest that rapamycin-dependent mTORC1 signaling is downstream of the membrane regulation mediating the firing behavior of CINs, and that this signaling pathway may not be directly involved in maintaining the basal phosphorylation levels intrinsically expressed in CINs. This latter point is of special importance since it implies that alternative signaling events may be responsible for the S6rp phosphorylation tone found in basal conditions. Certainly, rapamycin- and/or mTORC1-independent pathways such as the PKC, the MAPK or the cAMP/PKA pathways have been found to lead to S6rp phosphorylation in non-neuronal [23], [35]–[37] as well as neuronal systems [18], [38]. On the other hand, the requirement of the mTORC1 pathway under stimulated rather than basal firing conditions suggests that this cascade may be recruited when plasticity processes are initiated, as has been shown to occur in long-lasting synaptic plasticity processes in other neurons [25]. Although there is limited evidence for plasticity restricted to striatal interneurons [39], several studies have indeed demonstrated the existence of long-term changes in the intrinsic excitability of striatal CINs in response to synaptic stimulation [40]–[42]. The possibility that the mTORC1 signaling pathway, through direct regulation of protein translation processes, constitutes the molecular basis for long-term synaptic plasticity in striatal CINs is a tantalizing hypothesis that needs to be specifically addressed. Identification of the molecular events leading to basal vs. stimulated phosphorylation of S6rp may help explain the neurochemical origin of the sustained intrinsic firing of CINs, as well as the mechanisms leading to their neuronal adaptations in response to stimulatory conditions.

Despite the results reported here, the biological significance of S6rp phosphorylation in CINs remains to be elucidated. One of the better-described functional implications of S6rp is the regulation of cell size, which has been demonstrated in very different cellular systems [14], [15], [43]. Striatal giant CINs supply intense acetylcholine neuromodulation to virtually all striatal cells [44], a function that may impact on their metabolic rate over other types of neurons. It is tempting to speculate that the intense metabolic activity to which these neurons are subjected may explain their size, and that the phospho-S6rp signal is part of the important protein translation programs activated in these neurons in order to maintain their intrinsic firing properties as well as enzymatic turnover for acetylcholine homeostasis. Further studies contrasting the extent of S6rp phosphorylation with neuronal size and morphology will be required to explore this possibility.
